# Efficacy of Intravenous Immunoglobulin in Eliminating De Novo Donor-Specific Antibodies After Lung Transplantation: Importance of Early Intervention

**DOI:** 10.3389/ti.2025.15350

**Published:** 2025-11-03

**Authors:** Maximilian Vorstandlechner, Philip Degenfelder, Gökce Yavuz, Olaf M. Glueck, Julia R. Kovács, Julia Walter, Andrea Dick, Sebastian Michel, Christian P. Schneider, Michael Zoller, Jürgen Barton, Teresa Kauke

**Affiliations:** ^1^ Division of Thoracic Surgery, LMU University Hospital, Ludwig-Maximilians Universität (LMU) München, Munich, Germany; ^2^ Department of Medicine V, LMU University Hospital, Ludwig-Maximilians Universität (LMU) München, Munich, Germany; ^3^ Laboratory for Immunogenetics, Division of Transfusion Medicine, Cell Therapeutics and Haemostaseology, LMU University Hospital, Ludwig-Maximilians Universität (LMU) München, Munich, Germany; ^4^ Department of Cardiac Surgery, LMU University Hospital, Ludwig-Maximilians Universität (LMU) München, Munich, Germany; ^5^ Comprehensive Pneumology Center Munich (CPC-M), German Center for Lung Research (DZL), Munich, Germany; ^6^ Transplantation Center, LMU University Hospital, Ludwig-Maximilians Universität (LMU) München, Munich, Germany; ^7^ Department of Anesthesiology, LMU University Hospital, Ludwig-Maximilians Universität (LMU) München, Munich, Germany

**Keywords:** lung transplantation, dnDSA, AMR, ACR, CLAD

## Abstract

The development of de novo donor-specific anti-HLA antibodies (dnDSA) after lung transplantation (LuTX) has been increasingly linked to the onset of antibody-mediated rejection (AMR), chronic lung allograft dysfunction (CLAD), and impaired long-term outcomes. However, the therapeutic impact of intravenous immunoglobulin (IVIG) therapy in patients with dnDSA remains unclear. We conducted a retrospective single-center study of LuTX recipients (2015–2019) who developed dnDSA post-transplantation and received IVIG-based therapy. Patients were classified as responders or non-responders based on post-treatment antibody clearance. Clinical, immunological and functional outcomes were compared. Among 47 patients with dnDSA and IVIG-based therapy, 23 (48.9%) achieved complete antibody elimination. Preemptive treatment, defined as initiation of IVIG therapy before onset of clinical symptoms, was found to be an independent predictor of antibody clearance (odds ratio 29.5; p = 0.013). Responders showed significantly lower baseline MFI. While differences in CLAD-free survival favored responders, they did not reach statistical significance. Preemptive IVIG therapy in asymptomatic dnDSA-positive LuTX recipients may enhance antibody clearance and reduce CLAD risk. These findings support early intervention strategies and underscore the need for prospective trials to define optimal therapeutic thresholds and timing.

## Graphical Abstract

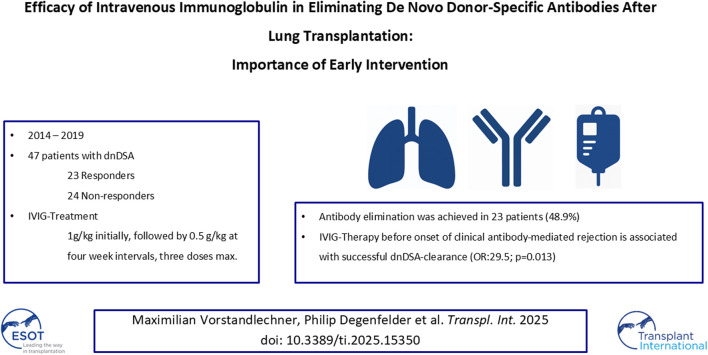

## Introduction

Lung transplantation (LuTX) remains the only definitive therapeutic option for patients with end-stage lung diseases (ELD) who have exhausted all other treatment modalities [1–3]. While short-term outcomes have improved markedly over recent decades, largely due to advances and standardization in immunosuppressive regimens and perioperative care, long-term survival remains critically constrained [4–6]. One of the principal challenges in this field is the complex immunological interplay between donor and recipient, which manifests in various forms of allograft rejection and contributes significantly to graft failure and patient mortality [3, 7, 8]. Acute cellular rejection (ACR) is a well-recognized complication during the first year following transplantation. Although typically responsive to corticosteroid therapy and not often fatal in the acute phase, ACR has been linked to increased long-term risk of developing chronic lung allograft dysfunction (CLAD), the predominant cause of late graft failure [9–12].

Beyond cellular rejection, humoral responses have gained recognition as major contributors to graft injury. The development of donor-specific anti-HLA antibodies (DSA) post-transplant (*de novo* DSA) is increasingly implicated in the pathogenesis of CLAD and reduced allograft survival [13–15]. Notably, the presence of dnDSAs has been associated with increased risk for both acute and chronic rejection, including the emergence of antibody-mediated rejection (AMR), a distinct and often insidious form of immune-mediated injury [16, 17]. The immunological basis for these processes lies in the human leukocyte antigen (HLA) system, a highly polymorphic set of genes that encodes the major histocompatibility complex (MHC) proteins. These molecules play an essential role in presenting antigens to T and B cells. Mismatches between donor and recipient HLA profiles are a potent trigger of alloimmune responses [18]. Formation of anti-HLA antibodies, whether preformed or *de novo*, can initiate a cascade of immune events involving complement activation, endothelial injury and eventual tissue destruction [19].

The diagnosis of pulmonary AMR remains difficult, in part due to its heterogeneous clinical presentation and the lack of definitive biomarkers. The consensus guidelines propose a multifactorial diagnostic framework involving the presence of DSAs, histological evidence of capillaritis, complement deposition (specifically C4d), evidence of graft dysfunction, and the exclusion of other etiologies [20]. Notably, subclinical AMR, defined by immunologic and histologic findings in the absence of functional impairment, is increasingly recognized as an early phase in the spectrum of humoral rejection [21]. Current therapeutic approaches for AMR are largely derived from treatment protocols in kidney and heart transplantation. These include intravenous immunoglobulin (IVIG), therapeutic plasma exchange (tPE), B-cell depletion with rituximab, and more recently, proteasome inhibitors (carfilzomib) and complement-blocking agents (eculizumab) [22–24]. Despite these efforts, treatment outcomes remain inconsistent. Studies have demonstrated variable antibody clearance rates and a persistently high risk of CLAD and death in patients with clinically manifest AMR [25]. Emerging evidence suggests that preemptive intervention in patients with newly detected dnDSAs, before the onset of overt graft dysfunction, may hold promise [21, 26, 27]. These findings suggest that early immunologic intervention could interrupt the pathogenic cascade that leads to chronic dysfunction, marking a potential shift in clinical strategy from reactive to preventive care.

Despite this progress, many critical questions remain unanswered. There is no consensus on the optimal treatment regimen, and randomized controlled trials are lacking. Furthermore, predictive markers that could guide patient selection and treatment decisions remain elusive. The significant heterogeneity in clinical response underscores the need for further mechanistic studies and controlled trials to identify which patients are most likely to benefit from specific therapeutic interventions. Ultimately, improving long-term outcomes in lung transplantation will depend not only on controlling cellular rejection, but on understanding and modulating the humoral immune response with greater precision.

## Materials and Methods

This retrospective single-center study was conducted at the Division of Thoracic surgery, LMU University Hospital, Munich. It was approved by the institutional review board of the faculty of medicine, Ludwig-Maximilians University Munich (UE No. 22-0123) and conducted in accordance with the ethical principles of the declaration of Helsinki. Only adult patients who underwent LuTX between 2015 and 2019 were eligible for inclusion. For the purpose of this study, only individuals who developed dnDSA postoperatively were included in further analyses. Follow-up included routine clinical controls and HLA antibody screening at standardized intervals: 1, 3, 6, 12, 18, 24, 30, 36, and 48 months postoperatively. Immunosuppression followed a standard triple-drug regimen consisting of corticosteroids, tacrolimus and mycophenolate mofetil.

All included patients had undergone routine pre- and post-transplant immunological monitoring. Genotyping was performed using sequence-specific oligonucleotide probes (SSO; LabType, One Lambda, Canoga Park, CA, United States), targeting HLA-A, -B, -C, DRB1, -DRB3/4/5, and DQB1 loci. Antibody screening and identification were performed using Luminex-based bead assays (LABScreen™, One Lambda), enabling high-sensitivity detection of HLA class I and II IgG antibodies. Mean fluorescence intensity (MFI) values greater than 1,000 were considered positive.

Within the dnDSA-positive cohort, patients were retrospectively categorized based on their immunological response to IVIG therapy. The standard therapeutic approach at our center for newly detected dnDSA is to initiate IVIG at 1 g/kg body weight, followed by three subsequent doses of 0.5 g/kg at 4-week intervals. The subset of patients who received IVIG-therapy, was divided into two groups: (1) those who achieved complete elimination of dnDSA following treatment (responders), and (2) those with persistent antibodies after therapy (non-responders). Complete antibody elimination was defined as the absence of previously detected donor-specific HLA antibodies in follow-up screenings, without subsequent recurrence.

Bronchoscopic surveillance was performed at regular intervals during the first 2 years post-transplant and subsequently based on clinical indication. Transbronchial biopsies (TBB) and histological diagnosis of ACR followed the International Society for Heart and Lung Transplantation (ISHLT) criteria, classifying rejection grades A0–A4 and lymphocytic bronchiolitis grades B0–B2R [28]. The 2016 ISHLT consensus report provided the basis for defining antibody-mediated rejection (AMR), distinguishing between clinical AMR, marked by detectable declines in lung function, which may occur without symptoms, and subclinical AMR, in which lung function remains preserved despite immunologic evidence of rejection [19, 20]. Lung function testing (forced expiratory volume in the first second, FEV1) was conducted in parallel with antibody assessments. To evaluate treatment efficacy, FEV1 measurements taken before therapy (baseline) were compared to post-treatment averages within 1 year after treatment initiation. CLAD was defined according to international consensus guidelines [29]. In this study, only bronchiolitis obliterans syndrome (BOS) was used as a clinical endpoint and diagnosed based on sustained FEV1 decline in the absence of alternative explanations [30]. Clinical, immunological, and procedural data were extracted from institutional records and the ET-database, including demographic information, transplant type, donor characteristics and intraoperative parameters (e.g., allograft ischemia time).

### Statistical Analysis

Statistical analyses were conducted using R (version 4.0.5) and RStudio (version 1.4.1106). Comparisons between groups were performed using the chi-square test or Fisher’s exact test for categorical variables and the Student’s t-test or Wilcoxon–Mann–Whitney test for continuous variables, as appropriate. Time-dependent outcomes were analyzed using Kaplan–Meier curves with log-rank tests. Logistic regression models were applied to identify factors independently associated with antibody elimination. Statistical significance was defined as a two-tailed p-value ≤0.05.

## Results

A total of 47 LuTX-recipients met the inclusion criteria and were enrolled in the analysis, among which 22 were female (46.8%) and 25 male (53.2%). The most common underlying disease was interstitial lung disease (ILD) (n = 21; 44.7%), followed by cystic fibrosis (CF) (n = 12; 25.5%) and chronic obstructive pulmonary disease (COPD) (n = 9; 19.1%). The median follow-up time post-transplantation was 18 months (range: 3–48 months). Antibody elimination was achieved in 48.9% of all patients (n = 23; responder), while 51% (n = 24; non-responder) exhibited persistent dnDSA throughout follow-up.

### Responders vs. Non-Responders

As depicted in Table 1, comparison of demographic characteristics between responders and non-responders revealed no statistically significant differences in recipient or donor age, sex distribution, transplant indication or surgical approach. Both groups had similar proportions of bilateral versus single lung transplants and comparable ischemia times.

**TABLE 1:** Non-Responders vs. Responders.

Non-responders and responders	Non-responders (n = 24)		Responders (n = 23)		*(p)*
	*(mean)*	*(sd)*	*(mean)*	*(sd)*	
Age (years)					
Recipient	51.2	12.1	46.8	15.2	0.28
Donor	44.7	16.9	48.8	15.6	0.4
Ischemia time (minutes)	522	125	510	128	0,75
	*(n)*	*(%)*	*(n)*	*(%)*	*(p)*
Sex					
Female	11	45.8%	11	47.8%	1,00
Male	13	44.2%	12	52.2%	
Underlying Diagnosis for LuTX					
ILD	11	45.8%	10	43.5%	1,00
COPD	4	16.7%	5	21.7%	
CF	6	25.0%	6	26.1%	
other	3	12.5%	2	8.7%	
One-year Survival	20	83.3%	21	91.3%	0.66
Lung Allograft Rejection					
ACR	8	33.3%	6	26.1%	0.75
Clinical AMR	16	66.7%	1	4.3%	<0.001
CLAD	7	29.2%	0	0.0%	0.009

ACR, acute cellular rejection; AMR, antibody-mediated rejection; CF, cystic fibrosis; CLAD, chronic lung allograft dysfunction; COPD, chronic obstructive pulmonary disease; LuTX, lung transplantation.

**FIGURE 1:**
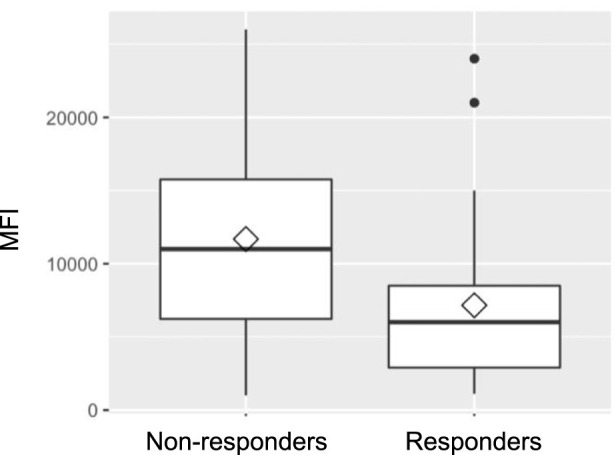
Mean fluorescence intensity (MFI).

When analyzing HLA class distribution of dnDSA, it became apparent that non-responders were more likely to develop HLA class II antibodies (95.8%; n = 23 vs. 78.3%; n = 18 in the responder group); however, this difference did not reach statistical significance (p = 0.097). As shown in Figure 1, MFI prior to therapy was significantly higher in the non-responder group compared to responders (mean 11,683 ± 7,055 vs. 7,152 ± 5,912; p = 0.019). Notably, the time to development of dnDSA was also significantly longer in non-responders than in responders (median: 94 days (IQR 35-343) vs. 39 days (IQR 22-65); p = 0.0025).

### Lung Function

**FIGURE 2:**
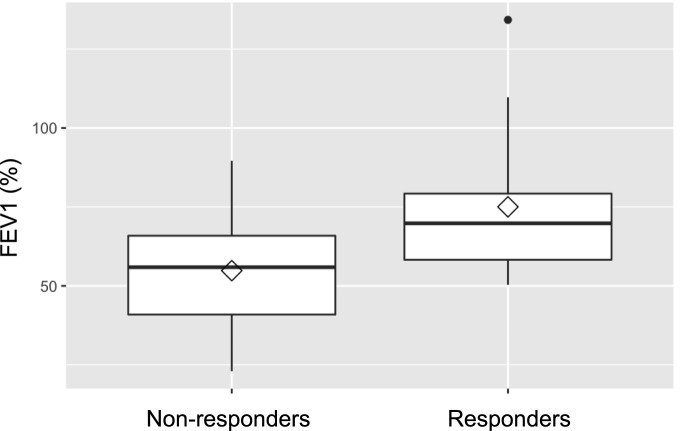
FEV1 pre IVIG-therapy.

**FIGURE 3:**
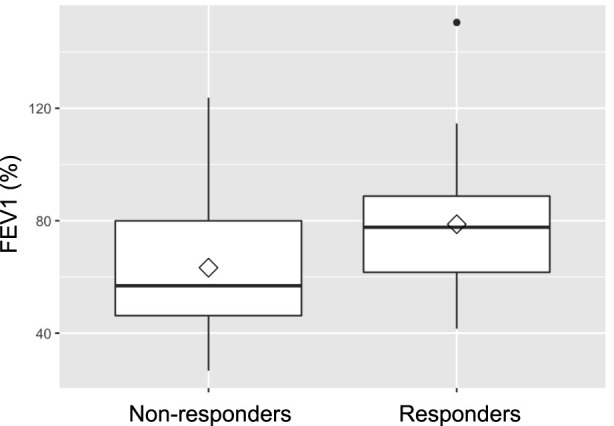
FEV1 1 year after IVIG-therapy initiation.

Baseline lung function, assessed by FEV_1_ prior to treatment, was retrospectively found to be significantly better in future responders (75.0% of predicted) than in non-responders (54.8%; p = 0.019). Lung function testing at 1 year following IVIG-therapy initiation, showed consistently higher FEV_1_ values (78.8% vs. 63.3%) in responders, although the difference between the groups was no longer statistically significant (p = 0.07). Figures 2, 3 illustrate FEV1 prior to and 1 year after IVIG-therapy.

### Acute Cellular Rejection (ACR)

ACR was identified in a total of 14 patients, corresponding to 29.8% of the entire study cohort. Among these cases, the majority were categorized as grade A1 rejection, which is characterized histologically by minimal perivascular mononuclear infiltrates without evidence of tissue injury. These milder rejection episodes were evenly distributed across both study groups (6/23 in the responder group vs. 6/24 in the non-responder group). Notably, a single episode of grade A2 rejection and one episode of grade A3 rejection were each observed and both occurred within the non-responder group. When comparing the incidence of ACR between treatment groups, no statistically significant difference was observed: 6 out of 23 responders (26.1%) experienced ACR, compared to 8 out of 24 non-responders (33.3%) (p = 0.75). These findings suggest that the rate of ACR was comparable between groups, regardless of treatment response.

### Antibody-Mediated Rejection (AMR)

**FIGURE 4:**
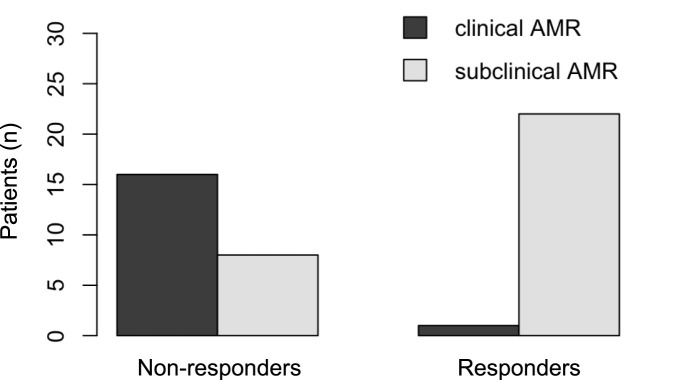
Antibody-mediated rejection (AMR) pre IVIG-therapy.

The prevalence of AMR at the time of IVIG initiation differed between responders and non-responders (Figure 4). For clinical AMR, only 1 of 23 responders (4.3%) met the criteria for possible clinical AMR, compared to 16 of 24 non-responders (66.7%). In contrast, subclinical AMR was more frequent among responders: 22 responders were classified with subclinical AMR at baseline, whereas only 8 non-responders fell into this category. Clinical AMR was therefore predominantly observed in non-responders, while subclinical AMR was more commonly seen in responders. Multivariable logistic regression analysis identified preemptive treatment, defined as initiation of therapy during subclinical AMR prior to the onset of clinical symptoms, as a significant independent predictor of dnDSA clearance (odds ratio 29.5 (OR); p = 0.013). Other variables assessed, including age, sex, MFI levels, antibody class, and timing of dnDSA detection were not significantly associated with antibody elimination.

### Chronic Lung Allograft Dysfunction (CLAD)

CLAD, defined according to standard criteria for BOS, was observed exclusively among patients in the non-responder group. A total of 7 non-responders (29.2%) were diagnosed with BOS during the observation period, whereas no responder developed this form of chronic allograft dysfunction. This difference was statistically significant (p = 0.009). Importantly, all cases of BOS were diagnosed prior to the initiation of IVIG therapy.

### Survival

One-year post-transplant survival in the overall cohort was 87.2%. Of the ten patients who died, eight were from the non-responder group and two from the responder group. Both deaths among responders were attributed to causes not directly related to the transplant (intracranial hemorrhage and cardiac failure). Although 1-year survival was numerically higher in responders compared to non-responders (91.3% vs. 83.3%), the difference was not statistically significant (p = 0.66). A similar trend was observed in 1-year survival following dnDSA detection: 91.3% in responders versus 71.4% in non-responders (p = 0.13). Survival 1 year after initiation of IVIG therapy was also higher in responders (90.9%) compared to non-responders (66.7%), although this difference did not reach statistical significance (p = 0.11). Kaplan–Meier analysis demonstrated non-significant differences in survival between groups following dnDSA detection (log-rank p = 0.11) and IVIG initiation (log-rank p = 0.06). Overall survival probabilty showed no differences between the two groups (log-rank p = 0.2).

## Discussion

The development of dnDSA following LuTX and their association with the onset of CLAD, impaired graft function and reduced survival have been well documented in numerous studies over recent years [13–16, 19, 31]. To date, the management of AMR remains heterogeneous, with no standardized protocols or universally accepted treatment strategies in place [21, 26, 32].

Our findings are consistent with growing evidence in literature that early immunological intervention can mitigate the development of chronic allograft dysfunction and extend graft survival [20, 33, 34]. While IVIG is well established in the treatment of AMR, its role in asymptomatic dnDSA or subclinical AMR is less clearly defined. We demonstrated that early intervention, particularly in asymptomatic patients with subclinical AMR, may significantly improve dnDSA clearance rates and associated clinical outcomes. Preemptive therapy, defined as the initiation of treatment before the onset of clinical symptoms, proved to be an independent predictor of successful dnDSA elimination in multivariable analysis (OR 29.5; *p* = 0.013). This observation aligns with data from Ius et al. (2018), who reported a 92% clearance rate following preemptive IVIG therapy in asymptomatic recipients, with graft survival comparable to DSA-negative controls [21]. Hachem et al. (2010) similarly found that preemptive therapy with IVIG solely or IVIG in combination with Rituximab resulted in DSA clearance in 65% of patients. Furthermore, patients witnessing successful antibody-depletion were less likely to develop BOS throughout the follow-up period [32]. Recent evidence presented by McDermott et al. provides important insight [27]. In a cohort of asymptomatic LuTX-recipients with dnDSA, preemptive IVIG monotherapy resulted in substantial reduction in antibody strength, with complete clearance in more than half of the patients. Moreover, they observe a trend toward lower rates of subsequent ACR among patients achieving DSA clearance.

The timing of dnDSA detection also appeared to influence treatment success. In our cohort, responders exhibited significantly earlier detection and received treatment sooner (median 15.0 vs. 36.5 days), suggesting that patients with early-onset dnDSA may benefit more from antibody-directed intervention. These observations are supported by Ensor et al. (2017) and Vacha et al. (2017), who reported that earlier therapy initiation was associated with improved antibody clearance and better clinical outcomes. Delayed treatment may permit maturation of antibodies and acquisition of complement-binding properties, which are known to reduce responsiveness to desensitization [24, 25].

While MFI values were not independently predictive of treatment success, we observed significantly higher pre-treatment MFI levels in non-responders, with the median nearly double that of responders. This observation is consistent with findings by [21, 24], who reported that lower baseline MFI values were associated with a higher likelihood of antibody clearance [21, 24]. In contrast, Timofeeva et al. (2021) did not find a significant correlation between pre-treatment MFI levels and overall survival; however, they observed that lower post-treatment MFI values following therapeutic plasma exchange (tPE) were significantly associated with improved survival [35].

Clinically, lung function and survival outcomes tended to be more favorable in the responder group. However, these differences were not statistically significant, which may be attributable to small sample size and ceiling effects, as many responders already had preserved baseline FEV_1_ values. Both groups showed only modest relative improvements in lung function and therapy in responders was more likely aimed at preventing decline rather than reversing damage. Similar associations between DSA elimination and improved BOS-free survival have been reported in other studies [24, 32, 35].

This study has several limitations that should be considered when interpreting the findings. First, the retrospective and single-center design inherently limits the generalizability of the results and may introduce selection or information bias. Although the study was conducted at a high-volume transplant center over a 5-year period, the overall sample size remained relatively small, thereby limiting statistical power and increasing the risk of type II error, particularly in subgroup analyses and survival comparisons. Furthermore, the classification of treatment response was based solely on dnDSA elimination, without histopathologic correlates such as C1q-binding capacity or tissue deposition. Due to institutional standards, uniform C4d staining was not performed, which may limit the ability to fully characterize the histopathologic phenotype of AMR and subclinical graft injury. However, given the limited sensitivity and specificity of C4d staining in lung allografts and the variability in its expression across AMR phenotypes, as highlighted in the ISHLT consensus report and subsequent studies, we consider its absence to have limited clinical impact in the context of this study [19]. While our study focused on IVIG monotherapy, the heterogeneity of dnDSA profiles and immune status across patients suggests that individualized or combination therapies may be required, an area not addressed in this analysis.

All cases of BOS in our cohort occurred prior to IVIG initiation and exclusively among non-responders, which may reflect inherent biological or immunologic differences between patients who did and did not achieve dnDSA clearance. The question of whether this observation strengthens or weakens the association between IVIG therapy and DSA clearance cannot be answered in the retrospective analysis and requires prospective data. This temporal pattern limits the ability to directly attribute the absence of BOS in responders to the effect of IVIG therapy.

Given the retrospective nature of the study, it did not include a control group of dnDSA-positive patients who did not receive IVIG therapy. This design inherently limits the ability to fully distinguish treatment effects from the natural course of dnDSA evolution. Future prospective studies with appropriate control cohorts will be important to confirm the therapeutic impact of IVIG. Lastly, the observational nature of the study precludes causal inference, and although multivariable analysis was performed, unmeasured confounders may still influence the associations observed.

Taken together, our findings highlight the importance of early, preemptive intervention in lung transplant recipients with dnDSA, particularly those who are asymptomatic and have moderate antibody burden. While high MFI and class II specificity may suggest reduced clearance probability, they should not preclude therapeutic attempts when clinical stability allows. DSA elimination remains a critical therapeutic goal, not only to limit immunologic injury but also to preserve long-term graft function and survival. Our findings underscore the need for early detection and individualized intervention in dnDSA-positive patients. Prospective multicenter trials are essential to validate risk-adapted treatment algorithms and define standardized thresholds for therapeutic initiation.

## Data Availability

The raw data supporting the conclusions of this article will be made available by the authors, without undue reservation.

## Abbreviations

ACR, Acute Cellular Rejection; AMR, Antibody-mediated Rejection; BOS, Bronchiolitis Obliterans Syndrome; CLAD, Chronic Lung Allograft Dysfunction; COPD, Chronic Obstructive Pulmonary Disease; dnDSA, *De Novo* Donor-Specific Antibody; DSA, Donor-Specific Antibody; ELD, End-Stage Lung Disease; FEV1, Forced Expiratory Volume in One Second; HLA, Human Leukocyte Antigen; ILD, Interstitial Lung Disease; IQR, Interquartile Range; ISHLT, International Society for Heart and Lung Transplantation; IVIG, Intravenous Immunoglobulin; LuTX, Lung Transplantation; MFI, Mean Fluorescence Intensity; MHC, Major Histocompatibility Complex; OR, Odds Ratio; SD, Standard Deviation; SSO, Sequence-Specific Oligonucleotide; TBB, Transbronchial Biopsy; tPE, Therapeutic Plasma Exchange.
