# Supplementary material for: Efficacy of Intravenous Immunoglobulin in Eliminating De Novo Donor-Specific Antibodies After Lung Transplantation: Importance of Early Intervention
Source: Transpl Int. 2025 Nov 3;38:15350. doi: 10.3389/ti.2025.15350 (PMC12620304; doi:10.3389/ti.2025.15350)
Supplement: Supplementary file 1 [file DataSheet1.pdf]

Supplementary Data

Patient ID	dnDSA class	dnDSA	peak MFI	response to IVIG-Therapy
01	II	DQB1*06:03	1000	non-responder
02	II	DQ2	8000	non-responder
03	I	A1	6500	non-responder
04	I, II	A3 DQ2	7000	non-responder
05	II	DQ7 DQ8 DQ9	10000	non-responder
06	II	DR14	10000	non-responder
07	II	DQA1*05:01	15000	non-responder
08	II	DQ7	8000	responder
09	II	DQA1*05:01	20000	non-responder
10	I, II	Cw 3 DQ5 DQ6	2000	responder
11	I, II	A32 B38 B44 DR7 DQ7	24000	non-responder
12	II	DQA1 *05:05	2000	non-responder
13	II	DQ6	3500	non-responder
14	II	DQ2 DP5	9000	responder
15	I, II	A1 DQ2	5700	responder
16	I, II	A2 Cw 6 Cw 12 DR7 DR15 DR51 DR53 DQ6	26000	non-responder
17	II	DQ8	12000	responder
18	II	DQ2	2300	responder
19	II	DPA1*02:01 DPA1*02:02 DQA1*05:01	22000	non-responder
20	II	DQ2 DR7	18000	non-responder
21	I, II	B8 DQA1 *05:01	15000	non-responder
22	I	B44	15000	responder
23	II	DQ5	6000	responder
24	II	DQA1 *05:01	3000	responder
25	II	DQ5	14000	non-responder
26	I, II	B62 Cw 10 DQB7 DR53	8000	responder
27	I	A2	2800	responder
28	I, II	Cw 5 DQ2 DQ8	21000	responder
29	II	DQ2	3000	non-responder
30	I	A2	6000	responder
31	II	DQ7	23000	non-responder
32	II	DQ7	5000	non-responder
33	I, II	B7 DQ7	11000	responder
34	I, II	A2 A23 B44 Cw 5 DQ2 DQ7	8000	responder
35	II	DQ5	6000	responder
36	II	DQ2 DQ6	8000	responder
37	II	DQ7	4000	responder
38	II	DQ7 DQ8	9000	responder
39	I	A2	1100	responder
40	II	DQ5	5000	responder
41	II	DQA1*05:01 DQ6	19000	non-responder
42	II	DQ5 DQ6	14000	non-responder
43	II	DQ7	2500	responder
44	I, II	B8 DQA1*05:01	12000	non-responder

45	II	DQ7	9000	non-responder
46	I, II	A31 DQ4	2100	responder
47	II	DQ7	5400	non-responder

dnDSA, de novo Donor-Specific Antibody; IVIG, intravenous immunoglobulin; MFI, mean fluorescence intensitiy
